# The Role of Individual Residues in the N-Terminus of Arrestin-1 in Rhodopsin Binding

**DOI:** 10.3390/ijms26020715

**Published:** 2025-01-16

**Authors:** Sergey A. Vishnivetskiy, Trishita Paul, Eugenia V. Gurevich, Vsevolod V. Gurevich

**Affiliations:** 1 Department of Pharmacology, Vanderbilt University, Nashville, TN 37232, USA; sergey.vishnivetskiy@vanderbilt.edu (S.A.V.); eugenia.gurevich@vanderbilt.edu (E.V.G.); 2 Department of Biomedical Engineering, Tulane University, New Orleans, LA 70118, USA; trishitapaul06@gmail.com

**Keywords:** arrestin, rhodopsin, mutagenesis, receptor binding

## Abstract

Sequences and three-dimensional structures of the four vertebrate arrestins are very similar, yet in sharp contrast to other subtypes, arrestin-1 demonstrates exquisite selectivity for the active phosphorylated form of its cognate receptor, rhodopsin. The N-terminus participates in receptor binding and serves as the anchor of the C-terminus, the release of which facilitates arrestin transition into a receptor-binding state. We tested the effects of substitutions of fourteen residues in the N-terminus of arrestin-1 on the binding to phosphorylated and unphosphorylated light-activated rhodopsin of wild-type protein and its enhanced mutant with C-terminal deletion that demonstrates higher binding to both functional forms of rhodopsin. Profound effects of mutations identified lysine-15 as the main phosphate sensor and phenylalanine-13 as the key anchor of the C-terminus. These residues are conserved in all arrestin subtypes. Substitutions of five other residues reduced arrestin-1 selectivity for phosphorylated rhodopsin, indicating that wild-type residues participate in fine-tuning of arrestin-1 binding. Differential effects of numerous substitutions in wild-type and an enhanced mutant arrestin-1 suggest that these two proteins bind rhodopsin differently.

## 1. Introduction

Arrestin-1 (We use systematic names of arrestin proteins, where the number after the dash indicates the order of cloning: arrestin-1 (historic names S-antigen, 48 kDa protein, visual or rod arrestin; SAG in HUGO database), arrestin-2 (β-arrestin or β-arrestin-1; ARRB1 in HUGO database), arrestin-3 (β-arrestin2 or hTHY-ARRX; ARRB2 in HUGO database), and arrestin-4 (cone or X-arrestin; ARR3 in HUGO database)) was the first member of the family discovered [1] and cloned [2]. Arrestin-1 directly competes with the visual G protein transducin [3,4] ensuring rapid and reproducible shutoff of rhodopsin signaling with sub-second kinetics in vivo [5,6,7,8,9,10,11]. After its first non-visual homolog was cloned [12] and shown to play a similar role in the signaling of the β_2_-adrenergic receptor [13], the paradigm of two-step homologous desensitization of the family of G protein-coupled receptors (GPCRs) was established: an active receptor is phosphorylated by a specific kinase, whereupon an arrestin protein binds to the active phosphoreceptor, shutting off its G protein-mediated signaling [14].

**Figure 1:**
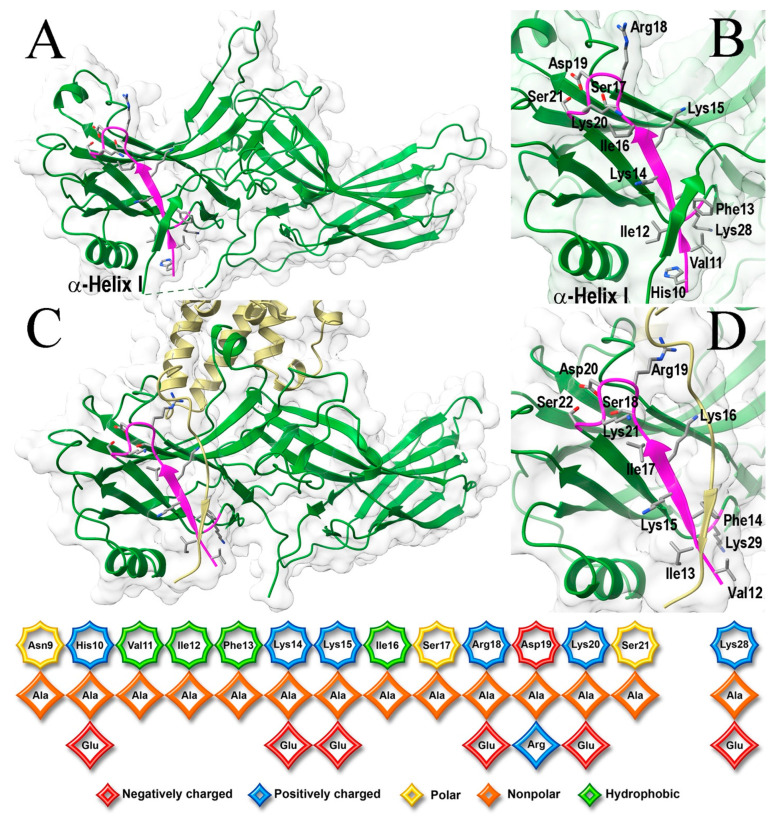
N-terminus of arrestin-1. (**A**) Crystal structure of basal bovine arrestin-1 (PDB ID 1CF1 [24]). (**B**) The enlarged area shows the residues targeted in this study. (**C**) Crystal structure of mouse arrestin-1-3A bound to rhodopsin (PDB ID 5W0P [30]). (**D**) The enlarged area of the complex shows the residues targeted in this study. In panels (**A**–**D**) mutated part is shown in magenta, with side chains of residues shown as stick models. The rest of the protein is shown in green. The surface of arrestin-1 is shown in pale gray. Rhodopsin in panels (**C**,**D**) is shown in yellow. Note the first Asn mutated here is not resolved in the structures and that the numbers in mouse arrestin-1 (labeled in panel (**D**)) are N + 1 relative to the bovine protein (labeled in panel (**B**)). The bottom panel shows the native sequence of the N-terminus of bovine arrestin-1 and the mutations introduced. All residues are color-coded as follows: small non-polar, orange; uncharged polar, yellow; positively charged, blue; negatively charged, red. Structural images in panels (**A**–**D**) were created in DS ViewerPro 6.0 (Dassault Systèmes, San Diego, CA, USA). Labels were added in Adobe Photoshop 2025 (Adobe, San Jose, CA, USA).

All arrestins bind active phosphorylated GPCRs with significantly higher affinity than unphosphorylated ones (reviewed in [15]). This makes biological sense: the time between the GPCR activation and phosphorylation provides a window of opportunity for G proteins, ensuring that each receptor activation event results in signaling. This is particularly important in rod photoreceptors that respond to single photons [16,17,18]. Arrestin-1 preferentially binds light-activated phosphorylated rhodopsin (P-Rh*) [19], demonstrating 10–20 times lower binding to the light-activated unphosphorylated form (Rh*) [20]. The ability of arrestin-1 to discriminate between the unphosphorylated and phosphorylated form of its cognate receptor is unmatched in the family [21]. Due to this unique feature, the “coincidence detector” model explaining how arrestins discriminate among functional forms of a receptor was first developed based on studies of arrestin-1 [20]. The model posits that the transition into a high-affinity receptor-binding state is triggered by the simultaneous engagement of two sensors, one recognizing receptor-attached phosphates and the other responding to active GPCR conformation [20]. This model appears to be valid for all arrestins [15]. The arrestin N-terminus plays a critical role in receptor binding: it contains a pair of lysines serving as the phosphate sensor [22,23] and the anchor of the C-terminus [24,25,26,27,28], the release of which accompanies arrestin binding to receptors [29,30,31,32,33,34,35,36,37]. Thus, a comprehensive analysis of this part of the arrestin molecule is necessary for two reasons: first, to identify specific structural features of arrestin-1 underlying its unique selectivity; second, to determine the functional role of residues conserved among arrestin subtypes that are likely involved in arrestin-receptor interaction and contribute to phosphorylation recognition. Here we tested the role of the arrestin-1 N-terminal residues in its binding to P-Rh*, with a particular interest in their role in its remarkable preference for this functional form of rhodopsin over Rh*, focusing on the part of the N-terminus conserved in evolution (positions 9–21 in bovine protein; Figure 1).

## 2. Results

**Figure 2:**
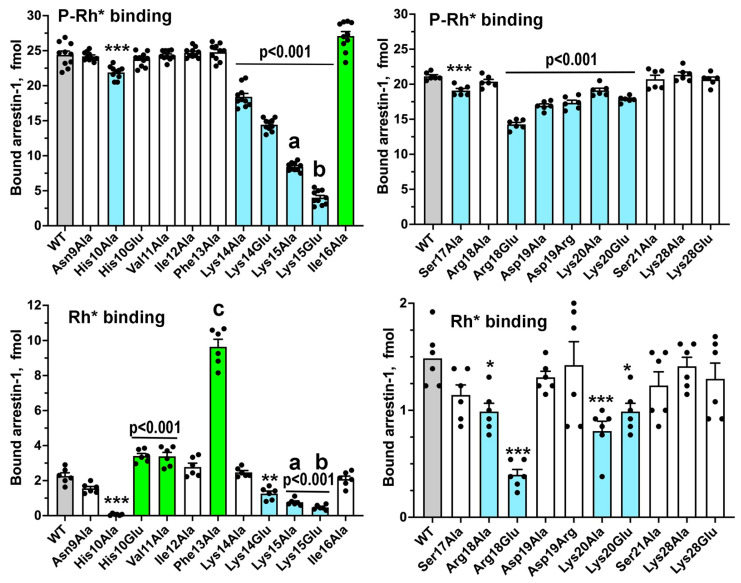
The effect of N-terminal mutations on WT background on arrestin-1 binding to rhodopsin. Specific binding of indicated mutants (fmol per assay) of arrestin-1 to P-Rh* and Rh* was determined using radiolabeled arrestins, produced in cell-free translation, in the direct binding assay with purified phosphorylated or unphosphorylated light-activated bovine rhodopsin, as described in Methods. Small black circles represent individual measurements (*n* = 6–8). The binding to P-Rh* and Rh* was analyzed separately in each of the two groups. Statistical significance of the differences between WT arrestin-1 and mutants was determined by one-way ANOVA followed by Dunnett post-hoc comparison to WT with correction for multiple comparisons. Statistical significance (*p* value) is either shown (directly applicable to all bars under the line) or indicated as follows: * *p*  <  0.05; **, *p* < 0.01; *** *p*  <  0.001 to WT; **a**, *p* < 0.0001 to Lys14Ala; **b**, *p* < 0.0001 to Lys14Glu; **c**, *p* < 0.0001 to all other mutants according to two-tailed Student’s *t*-test with Welch’s correction. Bars corresponding to mutants with increased or decreased binding are colored green and light blue, respectively; uncolored bars correspond to no significant difference from WT. Bars corresponding to WT are gray. Panels were created by Prism 10 (GraphPad Software, Inc., San Diego, CA, USA). The figure was assembled in Adobe Photoshop 2025 (Adobe, San Jose, CA, USA).

**Figure 3:**
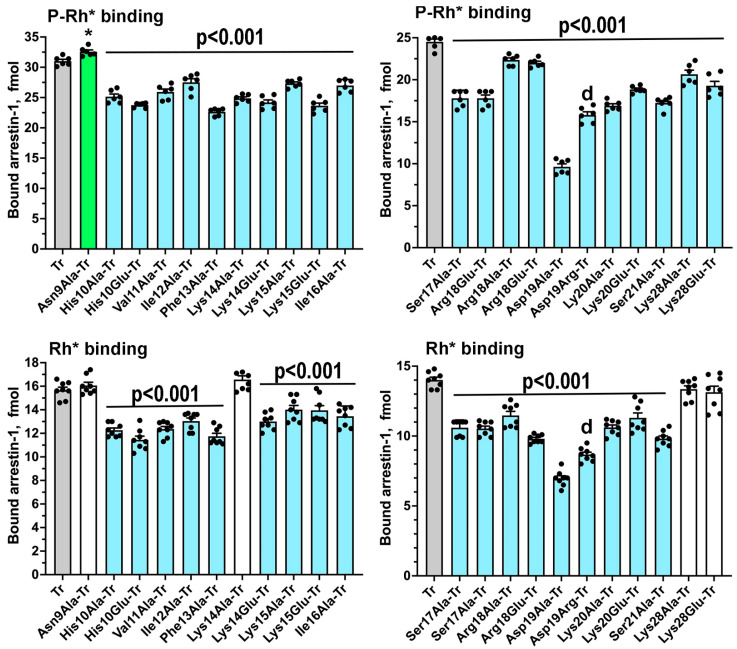
The effect of N-terminal mutations on Tr background on arrestin-1 binding to rhodopsin. The binding of indicated mutants to P-Rh* and Rh* was determined using radiolabeled arrestins, produced in cell-free translation, in the direct binding assay with purified phosphorylated or unphosphorylated light-activated bovine rhodopsin, as described in Methods. Specific binding is presented in fmol of bound arrestin-1 per assay. Small black circles represent individual measurements (*n* = 6). The binding to P-Rh* and Rh* was analyzed separately in each of the two groups. Statistical significance of the differences between the parental Tr and mutants was determined by ANOVA, followed by the Dunnett post-hoc test with correction for multiple comparisons. Statistical significance of the differences (*p* value) is either shown (applies to all bars under the line) or indicated, as follows: * *p*  <  0.05 to Tr; **d**, *p* < 0.001 to Asp19Ala, according to two-tailed Student’s *t*-test with Welch’s correction. Bars corresponding to mutants with increased or decreased binding are colored green and light blue, respectively; uncolored bars correspond to no significant difference from Tr. Bars corresponding to Tr are gray. Panels were created by Prism 10 (GraphPad Software, Inc., San Diego, CA, USA). The figure was assembled in Adobe Photoshop 2025 (Adobe, San Jose, CA, USA).

Here, we targeted the conserved part of the N-terminus, as well as Lys28, located near this element in the folded arrestin-1 in its basal and rhodopsin-bound conformation (Figure 1B,D). In addition to conventional alanine scanning, we introduced charge reversals of seven residues: six positively charged Lys, Arg, and His were replaced by negatively charged Glu; negatively charged Asp was replaced by positively charged Arg. It is entirely possible that the replacement of Asp with Lys, another residue carrying a full positive charge, or the replacement of positively charged residues with Asp instead of Glu, could have had a different effect. The total number of mutations tested was 21 (Figure 1, bottom panel). All mutants were tested on the background of WT bovine arrestin-1, as well as on the background of its C-terminally truncated form 1-378 (Tr) with enhanced binding to both P-Rh* and Rh* [20]. The functional importance of the targeted element is supported by the finding that the binding to P-Rh* and Rh* was significantly affected by 12 and 11 mutations, respectively, on the WT background (Figure 2), as well as by all 21 and 17 mutations, respectively, on the Tr background (Figure 3).

**Figure 4:**
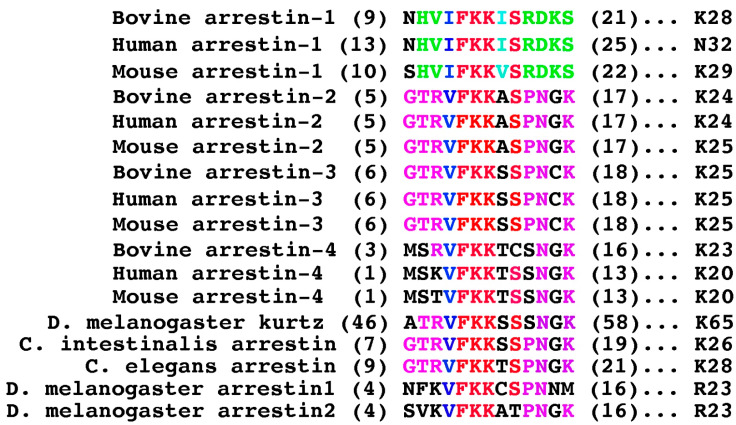
Conservation of the N-terminal sequence in arrestins. The numbers of the first and last residue in each arrestin are shown in parentheses before and after the sequence in single-letter code, respectively. Strictly conserved residues are shown in red, conservative substitutions in blue (light blue when the chemical nature of the residue is conserved only in arrestin-1 proteins), residues conserved only in arrestin-1 from different mammalian species are shown in green, and residues conserved only in the other subtypes, including arrestins from roundworm *C. elegans*, tunicate *Ciona intestinalis*, and fly *Drosophila*, are shown in magenta. Note that *C. elegans* and *C. intestinalis* have only one arrestin, whereas *Drosophila* has three: arrestin-1 and -2 are expressed in photoreceptors, and Kurtz is the non-visual subtype. The sequences are from: arrestin-1 bovine [2], human [40], mouse [41]; arrestin-2 bovine [42], human [43], mouse [44]; arrestin-3 bovine [42], human [45], mouse [44]; arrestin-4 bovine [46], human [47], mouse (GenBank AF156979); *Drosophila melanogaster* arrestin-1 [48], arrestin-2 [49], and Kurtz [50]; *Ciona intestinalis* arrestin [51]; *C. elegans* arrestin [52]. Created in Adobe Photoshop 2025 (Adobe, San Jose, CA, USA).

The N-terminal-most mutation, Asn9Ala, produced the smallest effect: a slight increase of the Tr binding to P-Rh* and nothing else (Figure 2 and Figure 3), consistent with the idea that the distal N-terminus, which faces away from bound receptors in all solved structures of the complexes [29,30,31,32,33,34,35,36,37,38], does not play a role in the arrestin-GPCR interaction. Functional roles of positively charged His10, Arg18, Lys20, and Lys28 were tested before only in an assay with relatively low sensitivity [39]. His10Ala reduced the binding to P-Rh* and Rh* on both backgrounds, whereas the effects of placing a negatively charged glutamate in this position were different: while the binding on the Tr background was negatively affected, His10Glu did not appreciably change P-Rh* binding but increased Rh* binding of WT arrestin-1 (Figure 2 and Figure 3). This suggests that His-10 contributes to arrestin-1 selectivity for P-Rh* by hindering the binding to unphosphorylated Rh* and that upon rhodopsin binding, its side chain is likely involved in H-bonding rather than in a charge-charge interaction. Arg18Glu mutation reduced the binding to P-Rh* and Rh* on both backgrounds, whereas Arg18Ala substitution was detrimental only for the binding of Tr (Figure 2 and Figure 3). A more severe effect of charge reversal suggests that in WT arrestin-1, this residue likely interacts with a negatively charged partner in rhodopsin. Its effect on unphosphorylated Rh* binding indicates that its partner is not a rhodopsin-attached phosphate but a negatively charged side chain of one of the rhodopsin residues (Glu or Asp). Lys20Ala and Lys20Glu mutations reduced the binding of WT and Tr arrestin-1 to both forms of rhodopsin (Figure 2 and Figure 3), also suggesting that the interaction partner of this lysine is not a phosphate on rhodopsin, which is present only in P-Rh*. Although a homologous residue in the mouse arrestin-1 mutant was found in the vicinity of one of the phosphates in P-Rh* in the structure of the complex [30], our data indicate that this lysine is not an important phosphate-binding residue in WT arrestin-1. Lys28Ala and Lys28Glu substitutions reduced only the binding of Tr to P-Rh*, suggesting that this residue does not play a significant role in the interaction of WT arrestin-1 with rhodopsin. The lack of conservation of this lysine in human arrestin-1 (Figure 4) is consistent with this conclusion.

Severe negative effects of the substitutions of Lys15, which were much stronger in WT arrestin-1 than in its Tr form, the binding of which is much less dependent on rhodopsin phosphorylation (Figure 2 and Figure 3), demonstrate that it is one of the key players in arrestin-1 transition into a high-affinity rhodopsin-binding state upon encountering phosphorylated rhodopsin. Substitutions of Lys14 were also detrimental, although less so than substitutions of Lys15 (Figure 2 and Figure 3). These data are consistent with the notion that this pair of lysines serves as the phosphate sensor of arrestin-1 [23]. The more important role of Lys 15 than that of Lys14 is consistent with the finding that in the complex of mouse arrestin-1 with rhodopsin, homologous Lys16 interacts with two rhodopsin-attached phosphates, whereas the preceding Lys 15 (homolog of bovine Lys14) does not, although in the structure it contacts negatively charged rhodopsin residue Glu341 [30]. The latter observation explains why Lys14 substitutions negatively affect the binding to Rh*. However, Lys15 substitutions demonstrate an even stronger negative effect on Rh* binding (Figure 2), suggesting that this lysine, in addition to phosphate binding, also interacts with a negative charge on rhodopsin other than a phosphate (e.g., an aspartic or glutamic acid).

Elimination of the bulky hydrophobic side chain in position 16 by the Ile16Ala mutation slightly increased the binding of WT arrestin-1 to P-Rh* and somewhat decreased the binding of the Tr form to both P-Rh* and Rh* (Figure 2 and Figure 3). Relatively small effects suggest that homologs of Ile16 do not play an important role in arrestin binding to receptors, consistent with the lack of conservation of the chemical nature of the residue in this position in different arrestin subtypes (Figure 4). The next residue, Ser17 in bovine arrestin-1, is conserved in the family (Figure 4). Its replacement with alanine was detrimental to the binding of WT and Tr arrestin-1 to both functional forms of rhodopsin (Figure 2 and Figure 3). In contrast, the alanine substitution of Ser21, which is not conserved (Figure 4), did not affect the binding of WT arrestin-1, although it somewhat reduced the binding of Tr (Figure 2 and Figure 3). Unexpectedly, elimination of the side chain of Asp19 and charge reversal at this position had negative effects on WT arrestin-1 binding to P-Rh*, but not Rh* (Figure 2), suggesting an important role of the WT aspartate in the high selectivity of arrestin-1 for P-Rh*. The negative effects of both substitutions of Asp19 on Tr background were significantly stronger (Figure 3). The finding that the Asp19Ala mutation in Tr was more detrimental than Asp19Arg (Figure 3) suggests that the side chain of this residue participates in hydrogen bonding, rather than in charge-charge interactions with rhodopsin.

While three consecutive hydrophobic residues (Val11-Ile12-Phe13 in bovine arrestin-1) were hypothesized to serve as an anchor of the arrestin C-terminus by crystal structures of all arrestin subtypes [24,25,27,28], as well as by the functional testing [53,54,55,56], the role of individual residues in this cluster was not investigated. As Phe13Ala mutation increases WT arrestin-1 binding to Rh* to a much greater extent than any other substitution (Figure 2), the Phe13 appears to be critical, Val11 is much less important, whereas Ile12 between these residues, the side chain of which points in the opposite direction in the β-strand 1 [24], does not play a significant role. This conclusion is consistent with strict conservation of Phe in homologous positions in other arrestin subtypes (Figure 4). The presence of bulky hydrophobic residue in the preceding position in all arrestins (Figure 4) also indicates its functional importance, although the data (Figure 2) suggest that this residue does not play a role in receptor binding. Interestingly, alanine substitutions of all three residues somewhat reduced the binding of Tr to both forms of rhodopsin (Figure 3). Conceivably, these hydrophobic residues play an important role in rhodopsin binding of the mutant, but not of the WT protein.

## 3. Discussion

Members of the arrestin family of proteins, like GPCRs, are present in all animal species [57]. Mammals express four arrestin subtypes [58]. Arrestin-1 binding to P-Rh* is necessary for quenching light-induced rhodopsin signaling with sub-second kinetics [5,7,59], which ensures exceptional time resolution of rod photoreceptors [60,61]. Arrestin-4 binds cone opsins, playing a similar role in the shutoff of light response in cone photoreceptors [62]. Although the expression of arrestin-1 in cones is ~50 times higher, functional analysis suggests that arrestin-4 is responsible for about half of the shut-off of cone opsin signaling [62]. Arrestin-2 and -3 are key players in the homologous desensitization of most non-visual GPCRs [14,63,64]. GPCRs are the largest family of signaling proteins in animals [65] responding to a variety of stimuli, from hormones and neurotransmitters to light, odorants, peptides, proteins, extracellular calcium, etc. [66]. Humans express ~800 different GPCR subtypes and ~30% of clinically used drugs target various GPCRs [67,68,69,70,71]. In rhodopsin, the inherent flexibility of the seven-helix GPCR core [72,73,74] is suppressed by the covalent binding of 11-cis-retinal, an inverse agonist, which ensures extremely low noise [61]. Exceptional selectivity of arrestin-1 for P-Rh* [21] also contributes to the proper function of rod photoreceptors. Thus, understanding the molecular mechanisms of arrestin-mediated regulation of GPCR signaling, as well as the structural basis of unique selectivity of the arrestin-1 branch of the family, is biologically important.

All arrestins are elongated, two-domain molecules with the C-terminus coming back from the C-domain and forming a strong hydrophobic interaction with the N-domain, in which the N-terminus and α-helix I participate [24,25,26,27,28] (Figure 1A). Numerous studies suggest that, as far as receptor binding is concerned, different elements in arrestin proteins serve two distinct functions. One is direct participation in the binding to cognate GPCRs. These residues tend to be exposed on the surface and in the complex interact with receptor residues. The other is to serve as “breaks” maintaining the basal conformation and preventing high-affinity binding of arrestins to the “wrong” functional forms of GPCRs, i.e., everything except active phosphorylated receptors. The residues serving this latter function tend to be buried in the basal state. Two intramolecular “clasps” were shown to hold arrestins in the basal conformation: the polar core between the two domains and the three-element interaction between β-strand I, α-helix I, and β-strand XX of the C-terminus [24,25,26,27,28,75]. The disruption of both was shown by modeling to be necessary for arrestin transition into receptor-binding conformation [76]. Destabilization of either of these interactions by mutagenesis increases arrestin binding to all functional forms of cognate GPCRs, including activated unphosphorylated receptors [22,23,53,54,55,77,78,79,80,81,82,83,84,85,86] (reviewed in [15]).

The first few N-terminal residues are not resolved in the structures of arrestin-1 [24] and other subtypes [25,26,27,28], as well as in receptor-bound arrestin-1 [29,30], -2 [31,32,33,34,35,36,37,87], and -3 [87], suggesting that this part does not have a preferred conformation in either basal or receptor-bound state. The length of the N-terminus (note the difference in the position of the starting residue in Figure 4) and the identity of the first residues in it are not conserved in evolution [57,58], suggesting that these features are unlikely to be important for the function shared by all arrestins, binding cognate GPCRs. The fact that in the arrestin-rhodopsin complex, the distal N-terminus faces away from the receptor [29,30] is consistent with this notion.

Although the role of the conserved part of the arrestin-1 N-terminus (Figure 1) in P-Rh* binding is not in doubt, only the effects of the substitution of the hydrophobic cluster (Val11Ala + Ile12Ala + Phe13Ala) hypothesized to maintain the basal conformation by anchoring the C-terminus to the N-domain and two-point mutations of presumed phosphate-binding residues (Lys14Ala and Lys15Ala) (the numbering corresponds to the bovine protein) were previously tested [22,53]. The role of individual hydrophobic side chains of residues 11–13 has not been elucidated. Lysines 14 and 15 were proposed to serve as the phosphate sensor in arrestin-1 [22,23] and non-visual subtypes [88], but the relative roles of individual lysines in this pair were not unambiguously established. The role of the other residues in the N-terminus was tested experimentally only using a relatively low sensitivity method [39].

**Figure 5:**
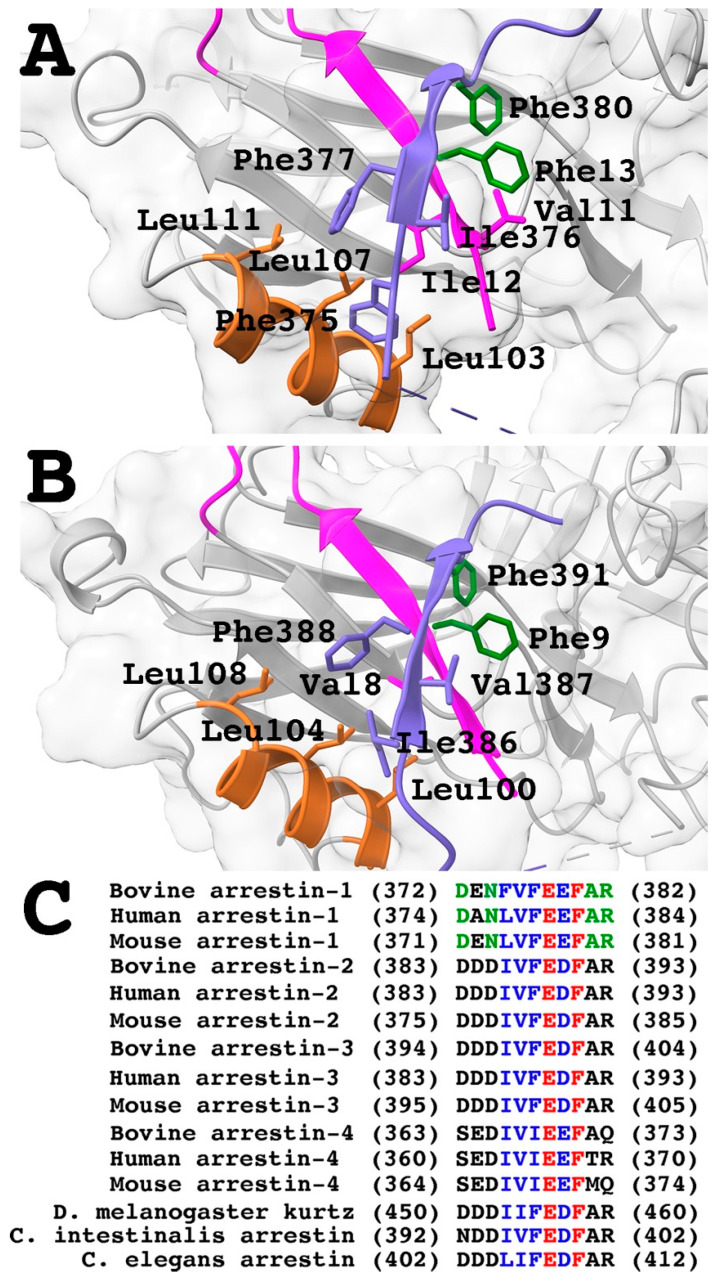
Three-element interaction in the basal conformation of arrestins. (**A**) Arrestin-1 (PDB ID 1CF1 [24]). The three parts participating in the interaction are colored as follows: the N-terminal β-strand I, including stick models of Val11 and Ile12, magenta; the α-helix I, including stick models of Leu103, Leu107, and Leu111, brown; the C-terminal β-strand XX, including stick models of Phe375, Ile376, and Phe377, navy blue; stick models of Phe13 of the N-terminus and Phe380 of the C-terminus, dark green. (**B**) Arrestin-2 (PDB ID 1G4M [25]). The three parts participating in the interaction are colored as follows: the N-terminal β-strand I, including the stick model of Val8, magenta; the α-helix I, including stick models of Leu100, Leu104, and Leu108, brown; the C-terminal β-strand XX, including stick models of Ile386, Val387, and Phe388, navy blue; stick models of Phe9 of the N-terminus and Phe391 of the C-terminus, dark green. (**C**) Sequence comparison of the C-termini of arrestins from various species (see legend to Figure 4 for the references). Strictly conserved residues are shown in red, conservative substitutions in dark blue, and residues conserved in arrestin-1 from different species in dark green. Structural images in panels (**A**,**B**) were created in DS ViewerPro 6.0 (Dassault Systèmes, San Diego, CA, USA), Labels in panels (**A**,**B**) were added and panel (**C**) was made in Adobe Photoshop 2025 (Adobe, San Jose, CA, USA).

Functional effects of the Phe13Ala mutation are typical for the destabilization of the basal conformation: a dramatic increase in the binding to the non-preferred form of rhodopsin, Rh* (Figure 1). The crystal structure of arrestin-1 showed that the hydrophobic cluster Val11-Ile12-Phe13 participates in the three-element interaction between β-strand I and α-helix I in the N-domain and β-strand XX in the C-terminus [24] (Figure 5A). A similar arrangement was revealed by the crystal structure of arrestin-2 [25] (Figure 5B). The structures of arrestin-1 (Figure 5A) and -2 (Figure 5B) suggest that phenylalanine in the N-terminal hydrophobic cluster interacts with phenylalanine in the C-terminus located after the cluster of three hydrophobic residues and two negative charges (Figure 5C). Note that different rotamers of corresponding phenylalanines were detected in different protomers of the crystal tetramer of arrestin-1 [75], suggesting that in solution, the two phenylalanines might be even closer to each other than in the structures shown. Importantly, phenylalanine homologous to Phe380 in bovine arrestin-1 is present in all arrestins, including the only isoform expressed by the roundworm *C. elegans*, suggesting that it appeared in evolution very early (Figure 5C). In fact, this part of the C-terminus of all arrestins shares the same motif: three bulky hydrophobic residues followed by two negative charges and phenylalanine after that (Figure 5C). Alanine substitution of the C-terminal Phe380 in arrestin-1 greatly increased the binding to Rh* [77], similar to the Phe13Ala mutation (Figure 2). This suggests that the interaction of the two phenylalanines plays an important role in anchoring the C-terminus to the N-domain. This explains why alanine substitutions of bulky hydrophobic residues in all three elements that disrupt this intramolecular “break”, or C-terminal deletion that has the same effect, increase the binding of all arrestins to their cognate receptors, phosphorylated and unphosphorylated [22,23,53,54,55,77,79,80,82,83,84,85,86].

Traditionally, it was assumed that the residues exposed on the arrestin-1 surface do not participate in inhibitory intramolecular interactions and are involved in rhodopsin binding, i.e., directly interacting with the receptor. This view was challenged by recent studies [85,86] revealing other functions: some residues specifically suppress the binding to Rh*, thereby increasing arrestin-1 selectivity for P-Rh*, and others participate in receptor binding of enhanced mutants but not of WT protein. Our experiments yielded several new insights into the role of individual N-terminal residues. Three appear to be largely irrelevant for rhodopsin binding of WT arrestin-1: Asn9, Ser21, and Lys28 (Figure 2). In contrast, His10, Ser17, Arg18, Asp19, and Lys20, which were not implicated in rhodopsin binding before, were found to participate in the interaction (Figure 2). The selective effect of Asp19 substitutions on the binding to P-Rh*, but not to Rh*, suggests that this aspartate likely interacts with its partner on rhodopsin only upon receptor phosphorylation. As negatively charged Asp cannot bind receptor-attached phosphates, this implies that the rhodopsin C-terminus (containing all phosphorylation sites) changes its conformation after the phosphates are attached so that Asp19 partner inaccessible in Rh* becomes accessible upon rhodopsin phosphorylation. This putative partner is not necessarily located on the C-terminus, as the conformational change in it would affect the accessibility of residues on other cytoplasmic parts of rhodopsin (i.e., three intracellular loops). In the crystal structure of the arrestin-1 complex with rhodopsin, this aspartate is located far from resolved rhodopsin elements [30]. However, the solved structure contained mutant human rhodopsin (two activating mutations, E113Q and M257Y, plus N2C and N282C to form a stabilizing disulfide bond absent in the WT protein) and fused mouse arrestin-1-(10-392) with triple alanine substitution in the C-terminus (L374A, V375A, F376A). The effects of Asp19 mutations suggest that the position of WT arrestin-1 relative to bound WT rhodopsin is not the same as in the solved structure. The structure of WT arrestin-1 bound to WT rhodopsin is necessary to test this hypothesis.

Previous studies suggested that the two lysines (Lys14 and Lys15 in bovine arrestin-1) strictly conserved in the family [58] (Figure 4) interact with rhodopsin-attached phosphates [22,23]. Our data identified Lys15 as the main phosphate sensor, whereas Lys14 appears to be less important (Figure 2). The three hydrophobic residues preceding these lysines in the linear sequence (Val11, Ile12, Phe13) were probed as a group, but not individually [53]. The crystal structure of arrestin-1 in its basal conformation [24] as well as functional evidence [53] shows that these residues, along with the three leucines in the α-helix I (Figure 1A), anchor the arrestin-1 C-terminus in the basal state. Alanine substitution of individual residues showed that Phe13 is a critical anchor, possibly assisted by Val11, whereas Ile12 does not play this role (Figure 2). Phenylalanine in position homologous to Phe13 is strictly conserved in the arrestin family [58] (Figure 4), suggesting that it plays the same role in different arrestin subtypes from various species. The preceding position is invariably occupied by a hydrophobic residue with a bulky side chain, but the first residue in this triplet is not conserved (Figure 4). Conceivably, Ile12 and its homologs in other arrestins are important for the folding and/or other functions, while the homologs of Phe13 are sufficient to anchor the arrestin C-terminus. The juxtaposition of the key phosphate sensor Lys15 and the key anchor of the C-terminus Phe13 (Figure 1) suggests the molecular mechanism whereby receptor-attached phosphate(s) facilitate the release of the C-terminus (anchored to the N-domain in the basal state of all arrestins [24,25,27,28]), which is the key event triggering arrestin transition into receptor-binding conformation [76]. Apparently, even a small shift induced by the phosphate interaction with Lys15 would move Phe13 out of position favorable for holding the C-terminus in place. As Phe13 is adjacent to phosphate-binding Lys14 and Lys15 (Figure 1), the effects of Phe13Ala mutations could reflect the impact of this substitution on the function of these lysines. However, in the crystal structures, this phenylalanine in free arrestin-1 contacts hydrophobic residue in the C-terminus [24,75] (Figure 5). Moreover, homologous phenylalanines in arrestin-2 [25,26], arrestin-3 [28], and arrestin-4 [27] in the structures of these proteins similarly interact with the hydrophobic residues in their C-termini. Thus, the most parsimonious explanation of the effects of Phe13Ala substitution is the disruption of the function documented in structures of basal arrestins, the anchoring of the C-terminus to the N-domain. Among natural amino acids, phenylalanine has the longest hydrophobic side chain. Structures suggest that an alanine with a very short side chain cannot perform this function. Our binding data identified seven residues in the arrestin-1 N-terminus (His10, Val11, Phe 13, Lys 15, Ser17, Asp19, and Lys20) that enhance its selectivity for P-Rh*. Importantly, four of these (His10, Val11, Asp19, and Lys20) are specific for the mammalian arrestin-1 proteins (Figure 4). Thus, this study identified several key N-terminal residues (Figure 2) that, along with previously identified residues [23,84,85,86], ensure the exquisite selectivity of arrestin-1 for P-Rh*.

As described so far, mutations in the human arrestin-1 gene are mostly inactivating due to premature termination [89,90,91,92]. The absence of functional arrestin-1 causes Oguchi disease (night blindness), although some arrestin-1 mutations also cause retinal degeneration (retinitis pigmentosa), likely due to the production of misfolded protein [93,94]. As the effects of N-terminal mutations described here are less drastic, it is likely that phenotypes are milder, so that humans carrying these mutations do not seek ophthalmological help. None of the mutants we used had folding problems (Supplementary Tables S1 and S2).

The most parsimonious explanation of our finding of significant differences in the effects of ten (out of 21 tested) point mutations (His10Glu, Val11Ala, Ile12Ala, Phe13Ala, Ile16Ala, Ser17Ala, Arg18Ala, Ser21Ala, Lys28Ala, and Lys28Glu) on rhodopsin binding of WT arrestin-1 and its Tr form (compare Figure 2 and Figure 3) is the hypothesis that WT arrestins and enhanced mutants bind GPCRs differently [23,84,85,86]. Significant differences in the effects of mutations on these two backgrounds in other arrestin-1 elements described earlier [84,85,86] also support this hypothesis. This hypothesis can be directly tested only by obtaining the structure of WT arrestin-1 in complex with WT rhodopsin, which would likely require the use of two-protein fusion (as in solved mutant-to-mutant structure [29,30]) and/or mild cross-linking followed by the addition of suitable antibodies (as in the structure of rhodopsin complex with GRK1 [95]). Note that various mutants were used in virtually all structural work: arrestin-2 with activating polar core mutation Arg169Glu in structures with β1-adrenergic [35] and 5HT_2B_ serotonin [32] receptors, arrestin-1-(10-392) with triple alanine substitution in the C-terminus (that detaches it from the N-domain) with rhodopsin [29,30], arrestin-2 with homologous triple alanine substitution with neurotensin NTS1 [37] receptor, arrestin-2 with various deletions in the C-terminus with M2 muscarinic [36], V2 vasopressin [31], neurotensin NTS1 [37], and β1-adrenergic [38] receptors, etc. Functional analysis of arrestin-1 interaction with rhodopsin suggests that solved structures of the arrestin-receptor complexes utilizing enhanced arrestins and mutant receptors do not necessarily reveal how WT arrestins bind cognate WT GPCRs. Distance measurements between rhodopsin and bound arrestin-1 [29,30], as well as the in-cell study of arrestin-2 interactions with parathyroid hormone receptor PTH1R [96], show that the complex of the same arrestin with the same receptor has multiple shapes, only one of which is revealed by the structures. Direct binding assay reports the sum of all binding modes. Thus, detected differences between WT arrestin-1 and its Tr mutant (compare Figure 2 and Figure 3) do not mean that WT protein does not bind rhodopsin the way Tr does. The results show that in the case of WT protein, Tr-like binding mode is responsible for a smaller fraction of the population of complexes than can be detected by statistical analysis.

Functionally important residues are conserved in evolution. In the mammalian arrestin-1 proteins, 11 out of 14 residues tested are identical, with one additional conservative substitution (Ile vs Val after the pair of lysines) (Figure 4). Only two residues are not conserved, the homologs of Asn9 and Lys28. Indeed, their substitutions did not affect the rhodopsin binding of WT arrestin-1 (Figure 2). The sequences of the N-termini of human, bovine, and mouse arrestin-2 and -3 are significantly different from arrestin-1. The N-terminal regions of the two non-visual subtypes are virtually identical, with only a single difference: Gly in arrestin-2 corresponds to Cys in arrestin-3 (Figure 4). As Gly occupies this position in “ancient” arrestins (Figure 4), it is likely the original, whereas Cys is a specific acquisition of the arrestin-3 branch. This sequence is part of JNK-activating arrestin-3-derived short peptides [97,98]. Importantly, arrestin-2 [99,100], as well as homologous arrestin-2-derived peptide with Gly in this position, does not facilitate JNK3 activation in cells [98]. The sequence of cone-specific arrestin-4 from the three mammalian species is closer to that of arrestin-2 and -3 than to arrestin-1. This is likely one of the reasons why arrestin-4 is less selective for active phosphorylated receptors, similar to non-visual arrestin-2 and -3 [27]. The first three residues, Gly-Thr-Arg, are conserved in non-visual and “ancient” arrestins (magenta in Figure 4) but strikingly different in visual subtypes: mammalian arrestin-1 and -4 and arrestin-1 and -2 from *Drosophila* (Figure 4). The functional role of this element in non-visual subtypes remains to be elucidated. Four residues are conserved in virtually all arrestin proteins (red in Figure 4). The only exceptions are bovine arrestin-4, where Ser is replaced with its geometrical (but not chemical) analog Cys, and *Drosophila* arrestin-2, where the same Ser is replaced by chemically similar Thr (Figure 4). In addition, one substitution is conservative: Ile in arrestin-1 corresponds to Val in all other arrestins (blue in Figure 4). Interestingly, Phe, the residue preceding the two lysines, and the second residue after them, Ser, are conserved even in species far removed from vertebrates: roundworm *C. elegans*, tunicate *C. intestinalis*, and fly *Drosophila* (in Figure 4). All four conserved residues play important roles in receptor binding: main anchor of the C-terminus (Phe13), main (Lys15), and auxiliary (Lys14) phosphate sensors, as well as Ser17, which was implicated in arrestin-rhodopsin interaction for the first time in this study. Conservation of the phosphate sensor and the anchor of the C-terminus is consistent with the idea that all arrestins use similar molecular mechanisms of transitioning into receptor-binding conformation. Although arrestins are required for the shutoff of rhodopsin signaling in *Drosophila* photoreceptors [101], the binding of arrestin in flies is triggered by rhodopsin activation and does not require its phosphorylation [102,103,104]. The conservation of lysines that serve as phosphate sensors in vertebrate arrestins (Figure 4) suggests that negatively charged side chain(s) in fly rhodopsin become accessible upon its activation and “attract” arrestin the way the phosphates do in vertebrates. The structure of the complex of one of *Drosophila* visual arrestins with light-activated rhodopsin is necessary to test this hypothesis. The reason for the conservation of several other residues in arrestins from non-vertebrate species, the sequences of which are closer to mammalian non-visual subtypes than to arrestin-1 (Figure 4), remains to be elucidated. Our data suggest that Arg18, Asp19, and Lys20, which are conserved in mammalian arrestin-1 proteins, are necessary for the binding of rhodopsin: their substitutions significantly reduced P-Rh* binding (Figure 2 and Figure 3). In arrestin-2, -3, and -4, this sequence is replaced with Pro-Asn-Gly, Pro-Asn-Cys, and Ser-Asn-Gly, respectively. This element in *C. intestinalis* and *C. elegans* proteins is the same as in vertebrate arrestin-2, whereas, in *Drosophila* non-visual arrestin Kurtz and visual arrestin2, it is the same as in human and mouse arrestin-4; *Drosophila* arrestin1 has Pro-Asn-Asn sequence in this place (Figure 4). These species are very far removed from mammals, so sequence similarity suggests that only limited variability of functionally important elements is allowed by evolution. As these three residues play a role in the rhodopsin binding of arrestin-1 (Figure 2), it is likely that this element in other arrestins participates in their binding to cognate GPCRs. Its function, as well as the role of Gly vs. Cys difference between arrestin-2 and -3, needs to be tested experimentally.

An inevitable limitation of the study of arrestin-1 binding to rhodopsin is that there is no viable alternative to the direct binding assay. Conventional study of dissociation kinetics is made impossible due to the rapid loss of all-trans-retinal by light-activated rhodopsin, which thereby converts into opsin (phosphorylated opsin in case of P-Rh* decay). Arrestin-1 binding to opsin and phospho-opsin differs from the binding to Rh* and P-Rh*: it is much lower [20] and appears to involve different arrestin-1 elements (compare [39] and [105]).

To summarize, we showed that (1) four residues conserved in all arrestins are critical for receptor binding; (2) these four plus four additional residues in the N-terminus specific for mammalian arrestin-1 proteins enhance the selectivity of this subtype for P-Rh*; (3) presented the data suggesting that WT arrestin-1 and its enhanced mutants interact with rhodopsin differently.

## 4. Materials and Methods

*Materials*. Radiolabeled [γ-^32^P]ATP and [^14^C]leucine were from Perkin-Elmer (Waltham, MA, USA). Restriction endonucleases, Vent DNA polymerase, and Quick T4 DNA ligase were from New England Biolabs (Ipswich, MA, USA). Rabbit reticulocyte lysate was made in bulk by Ambion (Austin, TX, USA). SP6 RNA polymerase was expressed in *E. coli* and purified, as described [106]. DNA purification kits for mini (3 mL of bacterial culture), midi (50 mL), and maxi (100 mL) preparations were from Zymo Research (Irvine, CA, USA). All other reagents were from Sigma-Aldrich (St. Louis, MO, USA).

*Mutagenesis and plasmid construction*. For in vitro mRNA synthesis bovine arrestin-1 was subcloned into a modified pGEM2 vector (Promega; Madison, WI, USA) with “idealized” 5′-UTR that does not require a cap for efficient translation [106] between Eco RI and Hind III sites, as described [107]. Mutations were introduced by PCR. Two restriction sites, Eco RI preceding “idealized” 5′-UTR and Bam HI in the coding sequence of bovine arrestin-1 [108] were used to subclone mutant fragments generated by PCR. Mutations were confirmed by dideoxy sequencing (GenHunter Corporation, Nashville, TN, USA). Eco RI—Bam HI fragments (164 bp) containing mutations were excised from WT constructs and subcloned into pGEM2-based construct encoding arrestin-1-(1-378) (Tr) mutant with much higher binding to Rh* [77].

*In vitro transcription* was performed, as described in detail [106]. Briefly, the plasmid was linearized after the coding sequence and incubated (30 μg/mL, ~30 nM) for 90 min at 38 °C with 1.5 unit/μL of purified SP6 RNA polymerase in 120 mM HEPES-KOH, pH 7.5, 16 mM MgCl_2_, 40 mM DTT, 2 mM spermidine-HCl, 100 μg/mL acetylated BSA, 5 units/mL of Prime RNase inhibitor, 2.5 units/mL of inorganic pyrophosphatase with 3 mM of each nucleoside triphosphate (ATP, GTP, CTP, and UTP). Then the mix was cooled on ice and 0.4 volume of ice-cold 9 M LiCl was added. After 10 min incubation on ice precipitated mRNA was pelleted by centrifugation for 10 min at 5000× *g* at 4 °C. The pellet was washed with 0.7 mL of ice-cold 2.5 M LiCl, then with 1 mL of 70% EtOH (both washes are followed by 5 min centrifugation at the above conditions). The pellet was air-dried for 5–7 min, then dissolved in one reaction volume of DNase/RNase-free water by vigorous vortexing. A 10 μL aliquot was taken at this stage for measurement. Immediately after that 0.1 volume of 3 M sodium acetate, pH 5.0, and 3.3 volumes of ethanol were added to precipitate purified mRNA. This mRNA suspension is stable at −80 °C for many months. For translation, an appropriate aliquot was removed, and mRNA was pelleted by centrifugation, washed with 70% ethanol, air dried (5–10 min), and dissolved in the desired volume of DNase/RNase-free water.

*Cell-free translation* of produced uncapped mRNAs in 70% rabbit reticulocyte lysate (RRL) was performed, as described [20]. Briefly, mRNA (100–150 μg/mL) was translated at 22 °C for 2 h. Before incubation, the following reagents were added to indicated final concentrations: potassium acetate, 120 mM, creatine phosphate, 30 mM, Prime RNase inhibitor, 5 units/mL, cAMP, 5 mM, 19 unlabeled amino acids, 50 μM each, [^14^C]leucine, 50 μM. RRL contains sufficient amounts of endogenous ATP, GTP, magnesium, and spermine. The concentration of endogenous leucine in RRL was determined by the isotope dilution method and taken into account when calculating the specific activity of leucine. Upon translation 1 mM ATP + 1 mM GTP were added, and the mix was incubated at 37 °C for 10 min (run-off). The mix was cooled on ice and ribosomes and aggregated proteins were pelleted by centrifugation for 1 h at 600,000× *g* (Beckman TLA 100.1 rotor, 100,000 rpm). The concentration of radiolabeled protein was determined before and after centrifugation: 5 μL of 10-fold diluted translation mix or supernatant was spotted onto 1 × 1 cm square on Whatman 3MM paper, allowed to dry, incubated 10 min in 10% trichloroacetic acid on ice, then boiled 10 min in 5% trichloroacetic acid, dried, cut out, incubated in scintillation vial with 0.5 mL of 10% SDS, whereupon water-compatible scintillation liquid was added, and the radioactivity was measured by liquid scintillation counting (Tri-Carb; PerkinElmer, Waltham, MA, USA). The specific activity of produced arrestin-1 was calculated by multiplying the specific activity of leucine by the number of leucines in it. The concentration of arrestin-1 in the supernatant was calculated by dividing protein-incorporated radioactivity by the calculated specific activity of the protein.

*Preparation of different functional forms of rhodopsin* was performed as described [109,110]. Rhodopsin-containing disc membranes were prepared from bovine rod outer segments (ROS) [109]. Rhodopsin in ROS was phosphorylated by endogenous rhodopsin kinase, as described [110]. Briefly, purified ROS (0.5 mg/mL total protein) were sonicated and incubated in bright light at room temperature for 4 h with 3 mM ATP, 3 mM GTP (to dissociate transducin from rhodopsin), 5 mM MgCl_2_, 1 mg/mL BSA, and 8 μg/mL of 11-cis-retinal. After phosphorylation, membranes were washed with 0.1 M potassium phosphate buffer, pH 7.4, and resuspended in this buffer at 1 mg/mL. Rhodopsin was regenerated in a tube wrapped in foil by 30 min incubation at room temperature with 3 mg/mL BSA and 3× molar excess of 11-cis-retinal. Then another 3× molar excess of 11-cis-retinal was added, followed by 30 min incubation. Rhodopsin-containing membranes were pelleted in the dark (Sorvall RC5Bplus centrifuge, SS-34 rotor (Sorvall, Waltham, MA, USA), 20,000 rpm), resuspended in dim red light in 50 mM Tris-HCl, pH 7.5, at the desired concentration, and aliquoted. Aliquots were kept wrapped in foil at −80 °C. Rhodopsin and phosphorylated rhodopsin are stable in these conditions for many months and tolerate > 10 cycles of freezing and thawing.

*The direct binding assay* was performed, as described [20]. Briefly, 1 nM arrestin-1 (50 fmol, specific activity 10.9–12.9 dpm/fmol) in the translation mix from which ribosomes were removed by high-speed centrifugation was incubated with 0.3 μg of indicated functional forms of rhodopsin (P-Rh* or Rh*; kept in the dark and dispensed in dim red light before the assay) in 50 μL of 50 mM Tris-HCL, pH 7.4, 100 mM potassium acetate, 1 mM EDTA, and 1 mM DTT for 5 min at 37 °C under room light. After incubation, the samples were cooled on ice for 1–2 min and then bound to rhodopsin-containing membranes arrestin-1 was separated from free arrestin-1 and unincorporated [^14^C]-leucine present in the translation mix at 4 °C by gel-filtration on a 2-mL Sepharose 2B-CL column. Radiolabeled arrestin-1 eluting with rhodopsin was quantified by liquid scintillation counting (Tri-Carb; PerkinElmer, Waltham, MA, USA). Relatively small (<10% of the total) non-specific “binding” (likely reflecting arrestin-1 aggregation during the assay) was determined in samples without rhodopsin and subtracted.

*Data Analysis and Statistics.* Statistical analysis was performed by one-way ANOVA (analysis of variance) with post-hoc Dunnett’s comparison to WT or Tr, respectively, with correction for multiple comparisons using Prism 9 software (GraphPad, Boston, MA, USA). *p* values  <  0.05 were considered statistically significant and indicated directly when applied to several values or as follows: * *p*  <  0.05; **, *p* < 0.01; *** *p*  <  0.001. For pairwise comparisons, a two-tailed Student’s *t*-test with Welch’s correction was used.

## Data Availability

The data are presented in the manuscript. Raw binding data obtained in each experiment are available upon request.

## Supplementary Materials

The following supporting information can be downloaded at: https://www.mdpi.com/article/10.3390/ijms26020715/s1.
