# Supplementary material for: The Role of Individual Residues in the N-Terminus of Arrestin-1 in Rhodopsin Binding
Source: Int J Mol Sci. 2025 Jan 16;26(2):715. doi: 10.3390/ijms26020715 (PMC11765510; doi:10.3390/ijms26020715)
Supplement: Supplementary file 1 [file ijms-26-00715-s001.zip › ijms-3363525-supplementary.pdf]

Supplemental Table S1

Cell-free translation results for N-terminal mutants on wild type background.

Arrestin-1 protein, WT background	Yield of soluble protein, fmol/μl	% Soluble
WT	37.2	70.0
WT	46.2	74.7
Asn9Ala	69.8	79.0
His10Ala	81.8	76.9
His10Glu	82.4	83.7
Val11Ala	82.2	86.2
Ile12Ala	83.4	83.1
Phe13Ala	64.9	83.6
Lys14Ala	60.0	84.0
Lys14Glu	69.8	89.1
Lys15Ala	71.5	97.1
Lys15Glu	80.4	87.9
Ile16Ala	57.8	79.2
Ser17Ala	79.9	86.8
Arg18Ala	81.9	82.5
Arg18Glu	95.1	94.6
Asp19Ala	55.7	77.8
Asp19Arg	56.9	77.7
Lys20Ala	61.5	78.5
Lys20Glu	74.9	94.4
Ser21Ala	65.7	82.8
Lys28Ala	56.0	82.7
Lys28Glu	48.6	81.0

The incorporation of the [^{14}C]-leucine in translation mix is determined before and after 1 h centrifugation at 600,000xg (to remove ribosomes and aggregated proteins). The fraction of soluble arrestin-1 protein (% soluble) is calculated as the ratio of its concentration in the supernatant and in translation mix before centrifugation. Proteins with folding problems give low yields and low % soluble. Note that absolute yields are also affected by the quality of mRNA used (the use of older or lower quality mRNAs results in lower yields). As the mutants were translated in two batches, the data for WT arrestin-1 are shown twice.

Supplemental Table S2

Cell-free translation results for N-terminal mutants on Tr background

Arrestin-1 protein, Truncated background (1-378)	Yield of soluble protein, fmol/μl	% Soluble
Tr	43.2	65.0
Tr	55.9	64.0
Asn9Ala	54.7	67.0
His10Ala	76.7	88.0
His10Glu	82.5	87.0
Val11Ala	69.0	78.8
Ile12Ala	65.0	78.7
Phe13Ala	71.3	94.1
Lys14Ala	56.7	74.5
Lys14Glu	67.1	90.4
Lys15Ala	57.4	76.1
Lys15Glu	61.8	81.4
Ile16Ala	66.2	67.2
Ser17Ala	91.0	67.6
Arg18Ala	89.8	66.7
Arg18Glu	96.8	87.0
Asp19Ala	74.9	67.6
Asp19Arg	72.9	64.2
Lys20Ala	87.8	75.4
Lys20Glu	84.8	82.6
Ser21Ala	95.9	66.8
Lys28Ala	68.5	74.6
Lys28Glu	75.2	70.9

The incorporation of the [^{14}C]-leucine in translation mix is determined before and after 1 h centrifugation at 600,000xg (to remove ribosomes and aggregated proteins). The fraction of soluble arrestin-1 protein (% soluble) is calculated as the ratio of its concentration in the supernatant and in translation mix before centrifugation. Proteins with folding problems give low yields and low % soluble. Note that absolute yields are also affected by the quality of mRNA used (the use of older or lower quality mRNAs results in lower yields). As the mutants were translated in two batches, the data for Tr arrestin-1 are shown twice.
